# The impact of cultural differences in self-representation on the neural substrates of posttraumatic stress disorder

**DOI:** 10.3402/ejpt.v7.30464

**Published:** 2016-06-13

**Authors:** Belinda J. Liddell, Laura Jobson

**Affiliations:** 1 School of Psychology, University of New South Wales Australia, Sydney, Australia; 2 School of Psychological Sciences, Monash University, Clayton, Australia; 3 Monash Institute of Cognitive and Clinical Neurosciences, Clayton, Australia

**Keywords:** Culture, self-construal, self-representation, individualist, collectivisit, individualistic, collectivistic, posttraumatic stress disorder, PTSD, trauma, neurocircuitry, emotion, attention, memory, self, attachment, interpersonal

## Abstract

A significant body of literature documents the neural mechanisms involved in the development and maintenance of posttraumatic stress disorder (PTSD). However, there is very little empirical work considering the influence of culture on these underlying mechanisms. Accumulating cultural neuroscience research clearly indicates that cultural differences in self-representation modulate many of the same neural processes proposed to be aberrant in PTSD. The objective of this review paper is to consider how culture may impact on the neural mechanisms underlying PTSD. We first outline five key affective and cognitive functions and their underlying neural correlates that have been identified as being disrupted in PTSD: (1) fear dysregulation; (2) attentional biases to threat; (3) emotion and autobiographical memory; (4) self-referential processing; and (5) attachment and interpersonal processing. Second, we consider prominent cultural theories and review the empirical research that has demonstrated the influence of cultural variations in self-representation on the neural substrates of these same five affective and cognitive functions. Finally, we propose a conceptual model that suggests that these five processes have major relevance to considering how culture may influence the neural processes underpinning PTSD.

**Highlights of the article:**

- Cultural variations in individualistic-collectivistic self-representation modulate many of the same neural and psychological processes disrupted in PTSD.
- These commonly affected processes include fear perception and regulation mechanisms, attentional biases (to threat), emotional and autobiographical memory systems, self-referential processing and attachment systems.
- A conceptual model is proposed whereby culture is considered integral to the development and maintenance of PTSD and its neural substrates.

Research indicates that posttraumatic stress disorder (PTSD) is a universal phenomenon observed cross-culturally (Figueira et al., 2007; Foa, Keane, Friedman, & Cohen, 2009). However, it remains unknown as to whether the processes implicated in the development, maintenance, and treatment of PTSD are culturally similar. This limitation extends to our current understanding of the neural mechanisms underpinning PTSD. There is currently an impressive body of literature documenting the neural substrates of PTSD, yet there is very little empirical work investigating the impact of culture on these systems. The importance of considering cultural influences is strengthened by research emerging from the field of cultural neuroscience that clearly indicates culture modulates many of the same neural and psychological processes aberrant in PTSD. This review will first provide a focused overview of the current understanding regarding core neural mechanisms underpinning PTSD. Second, we will consider prominent cultural theories. Third, a summary of investigations into how culture modulates neural correlates relevant to the key processes affected in PTSD is presented. Finally, we will develop a model that can be used to guide future empirical work in the domain of PTSD.

## Neurocircuitry underpinning five key disrupted mechanisms in PTSD

Across the spectrum of PTSD psychopathology, there are five key affective and cognitive functions that have been repeatedly identified as being disrupted in PTSD and have major relevance to conceptualising how culture may influence the neural substrates of PTSD. These are: (1) fear dysregulation; (2) attentional biases; (3) emotional memory impairments; (4) self-referential processing deficits; and (5) attachment and interpersonal processing alterations.

### Fear dysregulation

PTSD is characterised by core disturbances in the neural balance between prefrontal regulatory systems over fear and arousal systems. The central neurocircuitry model of PTSD purports that hyperactivity within fear-processing networks (including the amygdala, insula, and hippocampus), coupled with reduced regulatory activity within medial prefrontal cortical (MPFC) regions and cognitive control centres (dorsolateral prefrontal cortex; DLPFC), results in an inability to control fear responses (see reviews by Hayes, Hayes, & Mikedis, 2012; Jovanovic & Ressler, 2010; Patel, Spreng, Shin, & Girad, 2012; Pitman et al., 2012; Shin & Liberzon, 2010). Functionally, disruptions to these neural systems in PTSD are reflected in a hypervigilance to threat (see ‘Attention biases’ section), compromised fear learning and extinction processes (Jovanovic & Ressler, 2010; Milad et al., 2009), and heightened stress sensitivity and poor regulation over strong negative emotional reactions (New et al., 2009). An alternative dysregulation model, whereby hyperactive medial prefrontal regions overmodulate fear networks, has also been proposed for a dissociative subtype of PTSD (Lanius et al., 2010). This neural pattern is associated with emotional withdrawal and numbing symptoms, deactivation of arousal systems, as well as depersonalisation and derealisation symptoms (Lanius, 2015; Lanius, Brand, Vermetten, Frewen, & Spiegel, 2012).

Aberrant fear extinction, perception, and regulation processes in PTSD may also be due to problems with contextual processing (Liberzon & Sripada, 2008). A striking feature of PTSD is that re-experiencing symptoms usually occur in a safe context, suggesting that those with PTSD have difficulty updating their contextual representations of the traumatic event (Hayes et al., 2011; Van Rooij, Geuze, Kennis, Rademaker, & Vink, 2015), as well as generalised context-processing deficits (Van Rooij et al., 2014). Dysregulation between MPFC and hippocampal networks, vital to healthy contextualisation of emotional events (Maren, Phan, & Liberzon, 2013), form the neural basis for poor extinction of fear memories in safe contexts in PTSD (Jovanovic, Kazama, Bachevalier, & Davis, 2012; Parsons & Ressler, 2013). Critically, augmented fear responses in PTSD may reflect poor utilisation of contextual information to appropriately modulate emotional and behavioural responses (Garfinkel et al., 2014).

### Attention biases

A central feature of the aetiology of PTSD is a hypersensitivity to trauma-related stimuli that generalises to all threat cues (Cisler & Koster, 2010), reflecting an overall inefficiency in attentional resource allocation (Suvak & Barrett, 2011). Three mechanisms are proposed to account for this attentional bias in PTSD: (1) a hyperactive threat detection system engaging the amygdala (El Khoury-Malhame et al., 2011); (2) problems disengaging from threat (Pineles, Shipherd, Mostoufi, Abramovitz, & Yovel, 2009), associated with diminished engagement of frontoparietal attention systems (Blair et al., 2013); and (3) heightened avoidance of threat (Aupperle, Melrose, Stein, & Paulus, 2012), associated with dysregulated ventrolateral prefrontal cortical (VLPFC) and dorsal anterior cingulate cortex (dACC) functioning (Fani et al., 2012). In reality, it's likely that all forms of attentional biases operate in PTSD, with a recent study demonstrating that attention bias variability is an important predictor of acute PTSD symptoms (Iacoviello et al., 2014).

### Emotion memory

The hallmark of PTSD is the re-experiencing symptoms that involve distressing and involuntary intrusive memories of the traumatic event (Brewin, 2015). Prominent models of PTSD suggest that intrusions stem from fragmented, perceptual-based representations of the trauma in memory (Brewin, 2011), resulting from a breakdown in the hippocampal–ventromedial prefrontal cortex (VMPFC) network and precuneus during the consolidation period following trauma exposure (Brewin, 2011). Impairments in these networks intersect with the systems governing dysregulated fear processing and learning, including the amygdala, interfering with consolidation of both trauma-related and new emotional memories.

Another important aspect to the remembering of trauma is a memory trade-off, with biases towards recall of centralised information and the “gist” of an event under conditions of high arousal (McGaugh, 2013), to the detriment of recalling peripheral or contextual information (Christianson, 1992; Labar, 2007). The encoding of memories that are gist-based and lacking contextual details is proposed to be associated with PTSD memory distortions (Hayes et al., 2011; Hayes, Vanelzakker, & Shin, 2012; Williams et al., 2007), which is governed by significant alterations in the functional pathway between the hippocampus and MPFC (Jin et al., 2014). Changes in amygdala functioning, alongside the fusiform gyrus, appear to be critically involved in enhancing memory for central details of aversive events (Kensinger, 2009), influencing the wider hippocampus–VMPFC memory network (Waring & Kensinger, 2011). Overgeneralisation of episodic memories has also been found to be associated with abnormalities in the connectivity between the hippocampus and the MPFC (Xu et al., 2012). We propose that pre-existing biases towards attending to central or gist-based versus contextual cues, which may have a cultural basis, may influence how traumatic events are encoded, consolidated, and later retrieved. This will be examined in the ‘Cultural differences in attention biases’ section.

### Self-referential processing

PTSD patients often exhibit disturbances in self-referential processing, namely the manner in which an individual evaluates the self in relation to others (Lanius, Bluhm, & Frewen, 2011). The self-memory system model poses that trauma represents a threat to one's core sense of self that is difficult to reconcile with autobiographical knowledge (Conway, 2005). Violations to self-understanding and worldview following trauma is reflected clinically in negative appraisals of the self, others, and the world (Ehlers & Clark, 2000), which could be influenced by cultural factors.

Altered self-referential processing in PTSD also appears to have a specific neural basis. In one functional magnetic resonance imaging (fMRI) study, PTSD participants were slower to respond to self-relevant statements and demonstrated reduced VMPFC activity when compared with healthy controls (Bluhm et al., 2011). In another study, Frewen et al. (2011) found that women with PTSD following abuse trauma showed abnormal neural response patterns in the pregenual region of the ventral anterior cingulate cortex (vACC) when viewing their face and listening to positive trait adjectives. This region of the brain is linked to self-referential processing, alongside other medial prefrontal regions (Northoff et al., 2006). Meta-analyses show that PTSD patients routinely activate the retrosplenial cortex, precuneus (Sartory et al., 2013), and posterior cingulate gyrus (Ramage et al., 2013) during trauma or negative information processing—regions that are also associated with self-referential processing (Northoff et al., 2006). The involvement of these neural systems in PTSD supports the notion that functional alterations following trauma is related to self-identity and self-concept (Brewin, 2011).

### Attachment and interpersonal processing

Trauma exposure can significantly disrupt the attachment and interpersonal processing systems that normally assist coping with difficult events (Charuvastra & Cloitre, 2008). Insecure adult attachment style can impact on the ability of attachments to regulate negative emotions and confidence in available social support (Cloitre, Stovall-McClough, Zorbas, & Charuvastra, 2008), which may be functionally related to reduced hippocampal cell density (Quirin, Gillath, Pruessner, & Eggert, 2010). Conversely, the presence of supportive attachment figures may be critical to recovery from PTSD. Research has demonstrated that attachment priming can attenuate attentional biases to threat in PTSD (Mikulincer, Shaver, & Horesh, 2006) and increase activity within safety networks during pain exposure in healthy individuals, including the VMPFC (Eisenberger et al., 2011)—the key region responsible for fear extinction, healthy emotion regulation, and memory functioning. Overall, evidence of the benefit of social support for recovery from PTSD is mixed, with models suggesting that PTSD erodes the ability to harness social support and attachments (Bryant, 2016). Furthermore, oxytocin—a neuropeptide with widespread targets including the amygdala and hippocampus and important for facilitating attachment in mammals (Meyer-Lindenburg, Domes, Kirsch, & Heinrichs, 2011)—has been found to impact on a variety of the behavioural, neural, and neuroendocrine dysregulations observed in PTSD (Olff, 2012). Findings suggest that oxytocin reduces the acute stress response (Pitman, Orr, & Lasko, 1993) and improves neural functioning in emotion regulation (Olff et al., 2015) and reward networks (Nawijn et al., 2016) in PTSD patients, and is currently being examined as a potential therapeutic agent (Olff et al., 2015).

There is also a growing interest in the role of interpersonal factors in regulating emotional responses, including extreme negative emotions that define anxiety and mood disorders (Hofmann, 2014). A recent socio-interpersonal model of PTSD highlights the importance of considering the impact of interpersonal processes on the posttraumatic experience (Maercker & Hecker, 2016; Maercker & Horn, 2012). It is proposed that relevant interpersonal processes can be situated on three levels; (1) the individual level, comprising social affective states that relate to others; (2) the close relationship level, which includes attachment, social support, and interpersonal interpretation of traumatic events; and (3) the distant social level, which represents culture and society. Alterations to interpersonal processing across these three levels are proposed to predict response trajectories to traumatic stress (Maercker & Horn, 2012). This draws on the wider mental health literature that highlights how sociocultural context affects the expression, evaluation, and understanding of mental health symptoms, including explanatory models, coping strategies, and help-seeking behaviours (Alarcon et al., 2009).

## Culture and self

Culture has been conceptualised as an information system which is shared by a group and transmitted across generations, allowing groups to survive and derive meaning from life events (Kitayama & Juang, 2013). There is a growing recognition in the psychological sciences that different cultural groups differ in ways of thinking, behaving, and engaging with the world (Henrich, Heine, & Norenzayan, 2010). The worldview of an individual is gradually shaped by ongoing engagement with the reinforced practices of their cultural group, affecting the psychological landscape of the self (Kitayama & Uskul, 2011).

Psychological work on culture has placed the self as central in formulating models, generating research questions, and theoretically integrating a vast array of empirical findings (Kitayama & Uskul, 2011). From these analyses, it has emerged that people in different cultures have strikingly different understandings of the self. In Western, individualistic cultures, the self is perceived as an independent, autonomous entity, which emphasises private internal aspects (i.e., thoughts, emotions) and aims to be unique, realise internal attributes, and promote personal goals. In contrast, collectivistic, non-Western cultures perceive the self as an interdependent entity that emphasises external, public aspects (such as social roles, relationships) and social harmony (Markus & Kitayama, 2010). Such culturally divergent self-construals have been found to impact the very nature of individual experience and modulate brain functions governing emotional well-being, thinking, and behaviour. Therefore, cultural variations in self-representation can be viewed as an important factor in preserving homeostasis in the brain and body (Cacioppo & Bernston, 2011).

It is noted that the present review is focused specifically on cultural differences in representations of the self and how such variations might influence the neural correlates of PTSD. Indeed, the field of cultural neuroscience itself, as will be outlined in ‘Cultural neuroscience evidence’ section, has been dominated by studies focused on cultural differences in self-orientation as it reflects a core framework for meaning-making in the social world that has cross-cultural relevance (Cross, Hardin, & Gercek-Swing, 2011; Oyserman, 2011). However, some commentators argue for a broader understanding of culture in experimental psychology and neuroscience (Cohen, 2009; Henrich et al., 2010). Indeed, it has been suggested that self-construal itself is not entirely represented by the independent–interdependent dichotomy but rather is a multifactorial construct (Harb & Smith, 2008). Other cultural dimensions have been established by pioneering work conducted in the context of organisational psychology, which include temporal focus, indulgence-restraint and high–low power distance (Hofstede, 2001). Cohen (2009) argues that religion, socioeconomic status, and region within a country are alternative factors to individualism–collectivism that critically drive cultural variations. Unfortunately, there is very little empirical work conducted with regard to how these various cultural constructs influence brain function. As such, while this review recognises the importance of considering other cultural dimensions in the context of PTSD, understanding how cultural variations in self-representation explicitly influences the neural substrates of PTSD represents an important first step (Oyserman, Coon, & Kemmelmeier, 2002).

## Cultural neuroscience evidence

An expanding evidence base challenges the pervasive assumption that basic cognitive, perceptual, behavioural, and emotional processes are governed by neural systems that function universally in all humans. Rather, cultural theories suggest that variations in the representation of cultural values may strengthen specific neural processes that diverge by culture, consolidating particular behavioural response patterns, cognitions, and affective tendencies (Han et al., 2013; Han & Northoff, 2008; Kitayama & Uskul, 2011; Markus & Kitayama, 2010). Cultural neuroscience and psychological research demonstrates that human information processing is fundamentally shaped by culturally derived self-representations that manifest at both the population and individual level (Oyserman, Novin, Flinkenflögel, & Krabbendam, 2014; Park & Huang, 2010). Neural and psychological processes involved in fear processing and regulation, attention, memory encoding and retrieval, self-referential attributions, and attachment style are modulated by variation in self-representation, reflecting the same five processes that have been identified as central to known neural substrates of PTSD. If culture influences the neural correlates underpinning the very processes proposed to be involved in PTSD, this may have significance for understanding the neural substrates of PTSD. Here, we review the cultural neuroscience literature relating to these processes.

### Cultural differences in fear neurocircuitry and regulation of negative emotions

Culture influences the bottom-up, automatic processing of emotion, including the perception of biological fear signals (Adams et al., 2010). Eye-tracking studies reveal that participants from individualistic cultural groups view faces using an inverted triangle pattern perceptual strategy, whereas collectivistic participants focused on the central/eye region, resulting in miscategorisation of fear and disgust faces (Jack, Blais, Scheepers, Schyns, & Caldara, 2009). Martinez, Franco-Chaves, Milad, and Quirk (2014) also found cultural differences in physiological arousal responses during the habituation phase of a fear-learning task, suggesting differences in orienting responses to novel stimuli. These studies indicate that cultural factors shape important automatic emotion perception processes that are generally considered to be universal and fixed (Jack, Garrod, Caldara, & Schyns, 2012).

Emerging research highlights the role of culture in modulating amygdala responsivity (Derntl et al., 2012). Of relevance to the processing of trauma, Chiao et al. (2008) demonstrated that enhanced amygdala activation to fear expressed by members of one's own cultural group when compared with fear expressed by members of another cultural group, reflecting the amygdala's sensitivity to detecting potential threat to self. Similarly, oxytocin may be involved in motivating in-group favouritism by facilitating not only the development of trust, empathy, and prosociality but also preferential treatment of in-group and denigration of out-group members (De Dreu et al., 2010; De Dreu, Greer, Van Kleef, Shalvi, & Handgraaf, 2011). These findings suggest that culture operates on the processing of fear, which may have implications for understanding the neural basis of traumatic stress reactions and recovery across cultural groups.

Contextual processing biases have been found to differ between cultural groups. For instance, collectivistic participants draw more on social-based contextual cues to make emotional judgements of target face cues relative to individualistic participants (Masuda et al., 2008). These findings highlight that culturally influenced schemas influence cognitive processes, affecting attention allocation to emotional situations and appraisals. Given those with PTSD have been found to have deficits in contextual processing and culture influences this contextual processing, questions arise regarding how PTSD and culture interact to influence the perception and evaluation of context.

Disruptions in emotional regulation play a pivotal role in PTSD. Ford and Mauss (2015) highlight that culture influences the employment of specific emotion regulation strategies and the physiological consequences of implementing these strategies, thereby shaping overall well-being. Cultural differences have been found to automatically influence preferred regulation strategy (Mauss, Bunge, & Gross, 2008) and the neural substrates of emotion regulation (De Greck et al., 2012). Research has shown that while suppression is linked with reduced well-being in those from individualistic cultural backgrounds, suppression is unrelated to, or even beneficial for, the functioning of those from collectivistic cultural backgrounds (see Ford & Mauss, 2015). This is attributed to the collectivist view of promoting social harmony by minimising the impact of personal distress on others through suppressing the exhibition of strong emotions. Moreover, culture plays a role in determining the preferred homeostatic emotional state that regulation strategies serve to maintain. For instance, individualist cultures prefer high-arousal positive affective states (e.g., excitement), whereas collectivists prefer low-arousal positive states (e.g., calmness) (Tsai, Knutson, & Fung, 2006). Another study found that collectivistic participants preferentially activated the ventral anterior insula (related to the autonomic modulation of internal homeostasis), whereas an individualist group engaged the dorsal anterior insula (visceral-somatosensory/control) during evaluation of social narratives (Immordino-Yang, Yang, & Damasio, 2014). These studies suggest that culture impacts on the experience and neural correlates of arousal and affective states. Further, it is suggested that cultural attributes could play a role in modulating the nature of disruptions to emotion regulation functioning pivotal in PTSD.

### Cultural differences in attention biases

Behavioural and cognitive studies have highlighted the role of culture in modulating perception of the visual environment (Goh & Park, 2009; Kitayama & Uskul, 2011). Behavioural data have shown that those from collectivist cultures are more likely to attend to contextual and holistic aspects of visual cues than those from individualist cultures, who tend to fixate on object salience, localised details, and central objects (Nisbett & Masuda, 2003; Park & Huang, 2010). Research has demonstrated that these cultural differences are reflected in neural activation patterns (Park & Hwang, 2010). In one study, individualistic participants demonstrated greater selective functioning within the fusiform face area during face processing (consistent with object perception biases) compared with collectivistic participants, who showed a trend towards enhanced “landmark” processing in the lingual gyrus to house stimuli (Goh et al., 2010).

Moreover, individualists demonstrate faster eye movements to objects (Chua, Boland, & Nisbett, 2005) and stronger engagement in frontoparietal attention control regions when performing a culturally non-preferred version of a spatial judgement (Hedden, Ketay, Aron, Markus, & Gabrieli, 2008) or global/local processing task (Liddell et al., 2015). These biases in attention allocation during encoding also impacts on memory (Gutchess & Indeck, 2009): collectivist participants have better memory for contextual information and display difficulty recalling centrally presented information when paired with different backgrounds (e.g., Nisbett & Masuda, 2003).

### Cultural differences in autobiographical memory

Cross-cultural research has repeatedly demonstrated systematic cultural differences in autobiographical remembering; individualists frequently provide self-focused accounts of specific, personal events when compared with those from collectivistic cultures, who tend to focus on general group activities, social interactions, and significant others (Jobson, Moradi, Ramimi-Movaghar, Conway, & Dalgleish, 2014; Ross & Wang, 2010). While studies have not examined the neural mechanisms underlying these findings, recent work investigating cultural influences on neural processes underpinning information processing challenge the assumption that brain processes underlying autobiographical memories are universally similar (Ross & Wang, 2010).

Studies have indicated that cultural variability plays a role in the consolidation and retrieval of trauma memories in PTSD (Jobson, 2009). For example, Jobson and Dalgleish (2014) found that the more the trauma memory reflected culturally appropriate remembering, the fewer the number of intrusions. However, other studies have recently demonstrated that trauma survivors with PTSD from different cultural backgrounds evidence similar disruptions in their autobiographical remembering (Jobson et al., 2014). Such findings highlight the need for further research to investigate the influence of culture on the neural correlates of autobiographical memory, as central to memory disruptions in PTSD.

### Cultural differences in self-referential processing

PTSD models emphasise that trauma fundamentally affects the neural basis of self-concept (Brewin, 2011), but the empirical basis for these assertions are largely based on an individualistic sense of self-meaning (Jobson, 2009). Self-representations of those from both individualistic and collectivistic cultures have been traced to the VMPFC (Ng, Han, Mao, & Lai, 2010). However, for those from collectivist cultures the idea of self also includes reflections of others, and consequently significant others are also represented in the VMPFC (Ng et al., 2010). Zhu, Zhang, Fan, and Han (2007) compared neural activity in collectivists and individualists when making judgements about the self versus a significant other (i.e., mother). They found that individualistic participants showed heightened activation in the regions of the MPFC and pregenual area of the vACC when making judgements about themselves, and collectivistic participants recruited regions of the MPFC when making judgements about themselves and their mothers. Another study found that collectivists showed stronger engagement of pain processing centres in the brain (dACC, anterior insula) when perceiving others in emotional distress (Cheon et al., 2013). These findings provide strong evidence that cultural values shape neural functioning of self-referential systems (Park & Huang, 2010). Therefore, the question arises whether culture modulates the specific neural basis underpinning disrupted self-referential processing in PTSD.

### Cultural differences in attachment and support

Attachment is an important regulator of emotions but cultural differences in support seeking can substantially influence how attachment figures are utilised to cope with stress (Sherman, Kim, & Taylor, 2009). To individualists, the role of others is often to provide self-validation, with freely chosen relationships a means for meeting individual goals. Conversely, for collectivists, others are essential to self-definition, with goals and motivations significantly shaped and dominated by the needs of others in the immediate family or community (Adams & Plaut, 2003; Markus & Kitayama, 1991).

Culture has the potential to modulate two key features of social support: the decision to seek social support and the impact of social support seeking on health and well-being (Sherman et al., 2009). Research has shown that when deciding whether to seek social support, collectivists have a greater awareness of their impact on close others, are more sensitive to relational constraints, and believe help-seeking can negatively affect group harmony (Sherman et al., 2009). By contrast, individualists seem to focus more on the problem requiring support (Kim, Sherman, & Taylor, 2008; Sherman et al., 2009; Taylor, Welch, Kim, & Sherman, 2007). Cultural differences can influence how beneficial social support is during a stressful experience. During the experience of a social stressor, individualists reported less distress and exhibited reduced cortisol release when provided with explicit support but had the opposite response during implicit social support (Taylor et al., 2007). Collectivists showed the complete converse pattern, demonstrating reduced distress and cortisol release when provided with implicit support (Taylor et al., 2007). These findings show that both the psychological and physiological benefits of social support are dependent on culturally tuned expectations related to self-representation.

There also appears to be a neurobiological and genetic basis for cultural differences in social support and attachment. Kim et al. (2010) investigated whether an oxytocin receptor gene (OXTR rs53576, a gene related to socio-emotional reactivity) was sensitive to preferred social input according to culturally specific relational norms. As seeking emotional social support in times of distress is normative in individualistic but not in collectivistic cultures, Kim et al. (2010) examined the interaction between cultural group (American vs Korean), distress, and OXTR genotype on support-seeking behaviour. American participants who were more distressed and possessed the GG–AG genotype (the G allele is associated with more prosocial behaviour relative to the A allele) were more likely to seek emotional social support from others relative to those with the AA genotype (Kim et al., 2010). In contrast, Korean participants did not differ significantly by genotype. Furthermore, another study found an interaction between culture, emotion, regulation tendencies, and OXCR expression: American participants with the GG genotype were less likely to use emotional suppression, but Korean participants with the same genotype were more likely to use emotional suppression (Kim et al., 2011). These findings suggest that *OXTR* rs53576 is sensitive to input from cultural norms regarding emotional regulation and social support seeking.

## Proposed model of cultural influences on the neural dynamics of PTSD

The contribution of pre-existing belief systems and prior knowledge has been recognised in many cognitive models of PTSD (e. g. Ehlers & Clark, 2000; Foa & Rothbaum, 1998; Park, 2010). However, the recognition of the specific role of sociocultural factors in the modulation of many aspects of PTSD, which underpin the formation of an individual's identity and core belief systems, has only been recently recognised. According to the socio-interpersonal model of PTSD, cultural factors represent a third level of influence that can impact the trajectory of posttraumatic reactions (Maercker & Horn, 2012). The “threat to the conceptual self” model of PTSD (TCS; Jobson, 2009) also highlights that cultural influences are vital contributors to the constitution of prior experiences that influence the impact of trauma. Consequently, the effect of trauma on the self, and subsequent PTSD symptoms, may be at least partly, culturally determined.

To date, these cultural-based models of PTSD (Jobson, 2009; Maercker & Horn, 2012) have focused on clinical and psychological factors affected by culture, neglecting the consideration of how culture might modulate the neural correlates of PTSD. Conversely, neural models of PTSD have not accounted for the potential contribution of sociocultural factors in the development and maintenance of PTSD. As such, current neurobiological and psychological models of PTSD are potentially limited in that the vast majority of scientific evidence mapping the neural and cognitive mechanisms of PTSD has been collected using Western, individualistic clinical samples (Chiao & Blizinsky, 2013). Some neural models of PTSD indirectly recognise the importance of sociocultural factors. For example, Liberzon and Spirada (2008) suggest that poor contextual processing is a core deficit in PTSD, and that sociocultural factors are important determinants of extant context (Maren et al., 2013). Moreover, this and several other models consider disruptions to the neural basis of the self as vital to PTSD (Lanius et al., 2011; Shin & Liberzon, 2010), but this has not been extended to account for cultural variations in self-representation.

Given the lack of direct attention paid to cultural factors in neural models of PTSD to date, this review attempts to synthesise common themes across these two disparate fields and present a new model for considering cultural influences on the neural substrates of PTSD. Predominant cultural neuroscience models (e.g., Kitayama & Uskul, 2011; Oyserman et al., 2014) argue that reinforcement of culturally appropriate behaviours shapes neural functioning, which in turn contributes to further strengthening cultural tendencies. We suggest therefore that cultural frameworks fundamentally shape the generation of and maintenance of the very homeostasis disrupted by trauma exposure, thereby affecting the manifestation of posttraumatic stress reactions. When homeostasis is critically disturbed by a traumatic event, the ensuing physiological (e.g., hyperarousal), emotional (e.g., uncontrollable fear), behavioural (e.g., defence responses or avoidant tendencies), interpersonal (e.g., attachment processes), and cognitive (e.g., attentional biases to threat; memory disruptions and depletion of cognitive resources) ramifications may be modulated by cultural influences in both the acute post-exposure phase and the long-term recovery phase. In this manner, culture may affect the neural systems altered by trauma exposure, including resultant disruptions to affective and cognitive functioning, moderating important aspects of the development and course of PTSD, as well as recovery pathways.

We propose a hypothesis-generating conceptual model that merges evidence from the study of the neural substrates of PTSD with cultural neuroscience insights. This model predicts that culture will impact on the five affective and cognitive functions disrupted in PTSD highlighted in this review. Table 1 provides a summary of what is currently known regarding both the neural and psychological processes underpinning these five processes in PTSD and how these same neural and psychological mechanisms are influenced by culture. Table 1 also offers testable suggestions for cultural differences in the neural processes affected in PTSD for each of the five processes that will need to be empirically investigated. For instance:

1. *Fear processing and regulation*. Neural substrates underpinning emotion dysregulation in collectivists with PTSD may manifest via different pathways due to cultural preferences to suppress emotional responses, which likely impact on manifestation of re-experiencing and avoidance symptoms. Threat neurocircuitry disruptions and arousal mechanisms may be modulated by culture in PTSD, as a function of the cultural relevance of the activating stimulus.
2. *Attention biases to threat*. Contextual processing biases amongst collectivistic cultural groups may influence how attentional biases to threat are engaged in PTSD. Pre-existing cultural biases towards attending to central versus contextual cues may influence how traumatic events are encoded and the employment of attentional control mechanisms during high-arousal states.
3. *Autobiographical memory*. Cultural differences in autobiographical remembering may impact on how traumatic experiences are encoded and remembered in the brain, affecting the phenomenology of intrusions and the memory distortions characteristic of PTSD. Culture may also influence the remembering of centralised versus peripheral aspects of trauma cues, with implications for the manifestation of PTSD symptoms.
4. *Self-referential processing*. Disruptions to the neural basis of self-concept in PTSD may be critically informed by pre-existing cultural differences in self-referential processing. For example, a collectivist group's orientation towards significant others may impact on how trauma experiences are reflected in the neural substrates of self-identity.
5. *Interpersonal processing*. Collectivistic cultural groups draw on different interpersonal models to support their social well-being compared with individualists. These dynamics may impact how interpersonal trauma is experienced, the neural systems that are affected by such trauma, and how social support might be harnessed to benefit recovery.

**Table:** Table1. A summary across five core affective and cognitive functions with regard to: a) disruption in PTSD and related neural correlates; b) neuroscientific evidence of the impact of culture; and c) synthesis and hypothesis-generating suggestions for how culture might influence the neural processes affected in PTSD

	Neural and psychological substrates underpinning PTSD	Neural and psychological mechanisms influenced by culture	Suggestions for cultural differences in the neural processes affected in PTSD
1. Fear processing and regulation	Threat neurocircuitry alterations: impairments in MPFC regulation over subcortical threat detection networks, including amygdala, hippocampus; Reduced capacity to regulate strong negative emotions; impaired fear learning and extinction; increased negative appraisals and cognitions; Impairments in MPFC and hippocampal functioning could reflect reduced contextual processing.	Amygdala responses to fear faces modulated by cultural differences in individualistic versus collectivistic self-representation; Collectivistic cultural groups demonstrate restricted eye tracking to faces and utilise contextual cues to inform appraisals of affective information; Suppression regulation strategies detrimental to functioning in individualistic groups but beneficial to functioning in collectivistic groups.	Threat neurocircuitry disruptions and arousal mechanisms may be modulated by culture in PTSD, depending on the cultural relevance of the triggering stimulus; Emotion dysregulation following trauma may be modulated by culturally preferred emotion regulation strategies; Impaired contextual processing in PTSD may differentially impact those from collectivistic cultures.
2. Attentional biases to threat	Inefficient attentional resource allocation; Problems with disengaging from threat, hypervigilance to threat, and avoidance of threat associated with dysregulated cognitive control–emotional neural systems; Involves interaction between amygdala, dorsal frontoparietal attentional control networks, and dysregulated VLPFC and dACC activity.	Collectivistic cultural groups perceptually biased to attend to context/background cues; Individualistic cultural groups attend to local, salient, central cues; Reflected in cultural differences in engagement in place versus object processing regions in the brain.	Pre-existing cultural biases towards attending to central or gist-based versus contextual cues may influence how traumatic events are encoded, and the employment of attentional narrowing processes during high arousal; Disruption to attentional shifts in PTSD may also be affected by cultural preference.
3. Autobiographical and emotional memory	Intrusive trauma-related memory results from a breakdown in connections between medial temporal lobe (hippocampus) and medial prefrontal cortex (VMPFC); Under conditions of high arousal, a memory trade off with enhanced memory for centralised gist-related content versus reduced recall of peripheral details is underpinned by interconnections in the emotional memory network (e.g., amygdala, hippocampus, fusiform gyrus, inferior frontal gyrus); Overgeneralisation of autobiographical memories which has been associated with the hippocampus and abnormalities of the MPFC.	Intrusions are diminished if trauma encoding is self-related in individualists or other-related in collectivists; Cultural differences in attention allocation impacts on memory for central versus peripheral events; Autobiographical memories are focused on general social interactions and others amongst collectivists; individualist's memories are biased towards self-related, specific episodes.	The genesis and content of intrusive memories in PTSD, and the neural processes underpinning this, may be fundamentally influenced by cultural differences in memory construction; Cultural differences in deployment of attentional resources may impact how trauma events are encoded, as well as consolidated and retrieved in PTSD, modulating the memory trade-off effect; While one study has shown similar disruptions to autobiographical memory between cultural groups with PTSD, there has been very little investigation of how autobiographical memories and related neural processes are impacted by collectivist biases towards context and others.
4. Self-referential processing	Disturbances to self-referential processing and identity in PTSD, reflected in dysregulated MPFC, precuneus, and retrosplenial cortical functioning, as well as disorganised connectivity within the default mode network.	Individualists are biased towards self; collectivists are biased to considering self in relation to others; Culture shapes self-regulation systems in the brain: reflected in MPFC function during self-versus-other referential processing and judgements; Culture also influences the neural substrates of empathy and social evaluation.	Disruptions to self-referential systems may be distinct in PTSD sufferers with collectivistic cultural backgrounds, reflecting predominant other-focusedness.
5. Interpersonal processing and attachment	Disruption to interpersonal processing and attachment relationships in PTSD; Attachment cues may assist in alleviating PTSD symptoms, including attentional biases to threat and alleviating stress and social pain.	Cultural differences have been shown in the benefit afforded by types of social support, impacting on both the psychological and physiological mechanisms of stress; Cultural differences have also been demonstrated in the nature of support seeking during stress.	Cultural factors may influence how attachment regulates attentional biases to threat; Cultural variations may impact on pathways to post-trauma recovery and the efficacy of various treatment strategies, such as group therapy or harnessing of social support.

## Conclusion

At present we have a growing understanding of the neural substrates of PTSD. However, this understanding is culturally limited (Foa et al., 2009), despite PTSD rates being high amongst ethnic minority groups (Norris, 1992), refugees and asylum seekers (Silove, Sinnerbrink, Field, Manicavasagar, & Steel, 1997), and in conflict-affected populations (Steel et al., 2009). Many of these cultural groups adhere to collectivistic views of the self. There has also been very little consideration of how well-evidenced cultural differences in the neural systems supporting perception, attention, emotion, memory, and interpersonal processing are differentially disrupted by trauma exposure and PTSD. In this review, we propose that cultural differences in the neural correlates of these affective and cognitive functions will impact on how trauma disturbs these systems, and the subsequent manifestation and progression of PTSD, as well as recovery pathways. We suggest that it is therefore critical to develop an evidence base to examine the intersection between PTSD and cultural neuroscience, and present a conceptual framework to stimulate investigations in this domain.
